# Refractive Results: Safety and Efficacy of Secondary Piggyback Sensar*™* AR40 Intraocular Lens Implantation to Correct Pseudophakic Refractive Error

**DOI:** 10.1155/2016/4505812

**Published:** 2016-05-30

**Authors:** Alahmady Hamad Alsmman Hassan, Khulood M. Sayed, Mohammed ElAgooz, Ashraf Mostafa Elhawary

**Affiliations:** Department of Ophthalmology, Sohag Faculty of Medicine, Sohag University, Sohag 82511, Egypt

## Abstract

In this study we evaluate the visual outcomes, safety, efficacy, and stability of implanting of second sulcus intraocular lens (IOL) to correct unsatisfied ametropic patients after phacoemulsification.* Methods.* Retrospective study of 15 eyes (15 patients) underwent secondary intraocular lens implanted into the ciliary sulcus. The IOL used was a Sensar IOL three-piece foldable hydrophobic acrylic IOL. The first IOL in all patients was acrylic intrabagal IOL implanted in uncomplicated phacoemulsification surgery.* Results.* Fifteen eyes (15 patients) were involved in this study. Preoperatively, mean log⁡MAR UDVA and CDVA were 0.88 ± 0.22 and 0.19 ± 0.13, respectively, with a mean follow-up of 28 months (range: 24 to 36 months). At the end of the follow-up, all eyes achieved log⁡MAR UDVA of 0.20 ± 0.12 with postoperative refraction ranging from 0.00 to −0.50 D of attempted emmetropia.* Conclusions.* Implantation of the second sulcus SensarAR40 IOL was found to be safe, easy, and simple technique for management of ametropia following uncomplicated phacoemulsification.

## 1. Introduction

Phacoemulsification with intraocular lens (IOL) implantation is one of the most frequently performed surgical procedures in our clinical life. In uncomplicated surgeries with no other ocular pathology patients and doctors both suggest excellent vision in a very short period. Advanced surgical techniques, new IOL designs, recent accurate biometry machines, and advanced methods of IOL power calculation allow most cataract patients to restore very good quality vision in a very short time after surgery [1, 2]. Postoperative refractive surprise with different degrees of ametropia is a common cause of patient dissatisfaction after nice uncomplicated phacoemulsification and remains an important and challenging issue for ophthalmic surgeons [3]. Surgical choices for subsequent correction of residual ametropia after cataract surgery include keratorefractive laser surgery as LASIK or PRK [4, 5], IOL exchange, and sulcus IOL using the piggyback technique which is accurate easy and simple technique [6, 7].

Piggyback IOLs were indicated in those cases with extreme ametropia, corneal abnormalities, and thin corneas or when there is no available excimer laser [8]. Gayton et al. achieved excellent refractive outcomes by selecting piggyback IOL powers based on SE refractions after cataract surgery without considering keratometry or axial length. Eyes with myopic refractions received a minus IOL equal to the refractive error; eyes with hyperopic refractions received a plus IOL equal to 1.5 times the refractive error [9, 10]. Habot-Wilner et al. described an alternative method for hyperopic eyes that adds 1.0 D to 1.4 times the refractive error [11]. Holladay et al. created a formula to calculate the appropriate power of a piggyback IOL in myopic eyes. These approaches have proven to be more predictable than Gayton's initial methods [12]. In the current study we are trying to provide our experience with secondary piggyback intraocular lens implantation of sulcus foldable hydrophobic acrylic (IOL) in unsatisfied patients with refractive ametropia following uncomplicated phacoemulsification cataract surgeries to detect its long-term safety, efficacy, and stability.

## 2. Patients and Methods

A total of 15 eyes (9 REs and 6 LEs) in 15 patients (8 women and 7 men) underwent secondary piggyback to correct refractive ametropia following uncomplicated phacoemulsification.

In all cases, the lens used for secondary piggyback in the ciliary sulcus was Sensar AR40 with the following specifications:

- its 3-piece hydrophobic acrylic foldable IOL delivered to the sulcus plane by Unfolded Emerald Implantation System through 2.8 mm,
- the power available from −10 to +30 diopters,
- overall diameter 13.5 mm,
- optic 6 mm made of acrylic with a constant 118.4,
- haptic modified C material: blue core polymethylmethacrylate (PMMA) monofilament with compressibility/10 mm: 228 mg.

Calculation of the dioptric power of the IOL needed to achieve emmetropia was done as follows.

In cases of residual myopia the same power was used and in cases of residual hypermetropia we used 1.5 times the residual refractive error.

Exclusion criteria were any ocular pathology like corneal opacity, iritis, glaucoma, retinal detachment, or previous retinal surgery or any complication in the previous surgery like vitreous loss or lens displacement.

Inclusion criteria were previous phacoemulsification with residual ametropia myopia or hypermetropia of 1.5 diopter or more in unsatisfied patient. Intrabagal 1st IOL was mandatory.

The current study was done in Sohag Faculty of Medicine and all cases were done under the same circumstances by the same surgeon; all patients received preoperative Vigamox (moxifloxacin hydrochloride ophthalmic solution 0.5%) one hour before surgery and mydriatics (tropicamide 1% and phenylephrine 10%) every 15 minutes for four times before the surgery. All patients were operated on under topical anaesthesia Benox (benoxinate hydrochloride) (0.4% of Sterile Ophthalmic Solution 10 mL) every 5 minutes 3 times before the surgery and immediately on operative theatre; clear corneal incision with keratome 2.8 was done, followed by injection of intracameral lignocaine and viscoelastic material in the anterior chamber and then insertion of the IOL in the sulcus by the Unfolded Emerald Implantation System. Lastly wash of the viscoelastic material was done and hydration of the wound edges to leave a water tight wound.

All patients were followed up 8 hours postoperatively and then on the second day, after one week, after one month, and every 3 months in the follow-up period.

The follow-up examination included assessment of visual acuity, refraction, intraocular pressure, slit lamp examination, and fundus examination.

## 3. Results

Table 1 has collected data for each case. Patients' demographic data is shown in Table 2.

The study evaluated 15 eyes from 15 patients: 7 (47%) were men and 8 (53%) were women; 9 (60%) were right eyes. Mean patient age was 58 ± 7.6 years (range 42–70 years).

Of the 15 eyes of secondary piggyback included in the study, 7 were performed to correct myopic surprises and 8 were performed to correct hyperopic surprises. The duration from the 1st operation ranged from 1 month to 30 months (mean = 11 months). The longest follow-up period after secondary piggyback was 36 months (case  7) and the shortest was 24 month (case  3). The mean follow-up period was 28.87 months (Table 1).

Preoperatively, mean log⁡MAR UDVA was 0.875 ± 0.22 (range: 0.30 to 1.18) and mean log⁡MAR CDVA was 0.186 ± 0.13 (range: 0.00 to 0.48). All patients had improved UDVA postoperatively.

**Figure 1:**
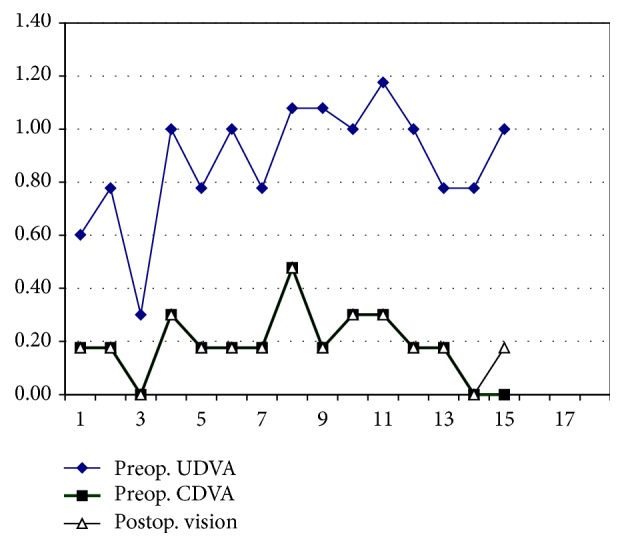
Pre- and postoperative vision in last visit.

At last follow-up, all eyes achieved log⁡MAR UDVA of 0.48 or better, with 2 eyes achieving log⁡MAR UDVA of 0 (Figure 1). One eye (case number 15) had postop. log⁡MAR UDVA less than preop. log⁡MAR CDVA due to faint after cataract. However the patient was happy and refused YAG laser posterior capsulotomy. No patient lost any lines of UDVA or CDVA except one, case  15 (Figure 1).

Preoperatively, mean spherical equivalent refraction was −0.23 ± 3.82 D ranging from 4.50 to −7.00 D. Postoperative mean spherical equivalent refraction was −0.19 ± 0.24 ranging from −0.50 to 0.25. In regard of the 2.8 corneal incision we made in all patients we noticed no astigmatic effect on final refraction of all patients.

**Figure 2:**
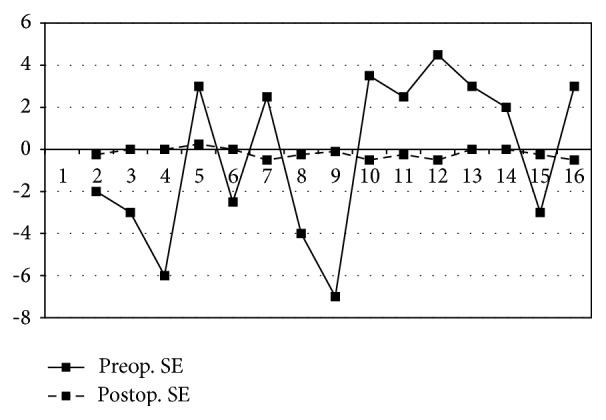
Pre- and postoperative refraction in last visit.

All patients were within 0.25 to 0.50 D of the attempted emmetropia, with 73% within 0.25 D (Figure 2).

Analysis of our data showed good correlation between the postoperative VA and both CDVA (*r* = 0.935) and age of the patient (*r* = 0.869). Moderate correlation exists between postop. VA and both UCVA and follow-up period (Table 3).

When all variables were entered in a regression model, the preoperative CDVA was the only predictor for postoperative vision (*R* ^2^ = 0.874) and the predictability was not strengthened by adding any other factor.

We did not encounter any intraoperative complications. None of the secondary IOLs needed to be repositioned at the end of surgery. No postoperative complications related to piggyback IOLs (e.g., pupillary block glaucoma and pigment dispersion syndrome) or interlenticular opacification were observed during the follow-up period. One eye (6.7%) had a slightly decentered IOL detected on the 1st postoperative day (case number 11) without a significant visual affection with a full patient satisfaction so recentration was not required. Otherwise, following dilation and retroillumination, all IOLs were well-centered and no cases of IOL rotation or tilt were observed. One eye (6.7%) had an IOP rise of 24 mmHg on the 1st postoperative day which was controlled with a topical B-blocker antiglaucoma medication. The IOP remained stable without treatment throughout follow-up.

Two eyes required YAG laser posterior capsulotomy for posterior capsular opacification (at 6 months and 1.5 years, resp.). At last follow-up, all patients were spectacle-independent for distance.

## 4. Discussion

Postoperative refractive surprises are usually due to placement of an incorrect power IOL as a result of a preoperative error in axial length measurement or keratometry [13].

The undesirable refractive errors after cataract surgery can be treated surgically in various ways [14], including corneal and limbal relaxing incisions, keratorefractive laser surgery, IOL exchange, or, more recently, implantation of a LAL [15].

Laser refractive surgery is effective and safe for the correction of residual refractive error. However, it could create potential complications that might be more common in older patients related to dry eye and wound healing [16].

Explanation of the original IOL followed by insertion of a new IOL with the correct dioptric power is a difficult procedure that entails a higher risk than other alternatives [14].

Relaxing incisions can correct low and moderate astigmatic errors but are less precise and can be complicated by placement on the incorrect axis, perforation, pain, and infection [15].

In 1993, Gayton and Sanders [17] reported the first piggyback IOL implantation, which was done to provide sufficient plus power in a microphthalmic eye. Others [9, 18, 19] subsequently used this approach more broadly for the correction of high hyperopia. In 1999, Gayton et al. [18] reported a case series demonstrating that piggyback IOLs could also be used to correct a wide range of pseudophakic ametropias. Gayton et al. [20] outlined several advantages of piggyback IOLs over IOL exchange.

In lens exchange procedure surgical correction should be done as soon as possible, before the formation of capsular adhesions with the IOL [14]. Implanting a second IOL anterior to the one already in place is generally easier and less traumatic than IOL exchange because it requires less manipulation. Another advantage of piggyback IOLs is the relative simplicity of IOL power selection [21].

Another important advantage of secondary piggyback versus IOL exchange is that it is not necessary to know the cause of the postoperative refractive surprise, that is, whether the error occurred during keratometry or biometry, in manufacturing the IOL, in using an inadequate formula for calculating the IOL, and so forth. All these issues become irrelevant to solving the problem, as the solution does not depend on knowing its cause [14].

The major drawbacks of piggyback IOLs are the risk for interlenticular opacities, the increased risk for IOL-related complications due to chafing against the pigment epithelium of the iris, the possibility of piggyback IOL dislocation due to the approximation of 2 convex surfaces, and the theoretical possibility of IOL curvature change from compression [21]. Such complications were not reported in our series nor in a similar study by Trindade with the single-piece PMMA-Slim*™* IOL [14]. Any of these complications can necessitate removal of both IOLs.

In contrast, Eleftheriadis et al. [22] recognized interlenticular opacification in a 50-year-old man who developed IOL bilaterally after piggyback AcrySof IOL implantation which necessitates the explanation of two pairs of AcrySof IOLs.

Importantly, an angulation should exist between the optic and haptic parts of the IOL. The edges of the optic part should preferably be rounded. All these recommendations aim to prevent complications that can occur after secondary piggyback in the ciliary sulcus [14].

In this study, the secondary IOL offered an effective method to correct unexpected postoperative visually significant refractive error. These results are consistent with Falzon et al.'s study who obtained nearly the same results with the Sulcoflex Intraocular Lens with no intraoperative and minimal postoperative complications [14]. All patients achieved a mean postoperative log⁡MAR UDVA of 0.20 ± 0.12 with no evidence of iris chafing, pigment dispersion, or interlenticular opacification indicating minimal interaction of the secondary IOL with the primary IOL and uveal tissues.

This study found that the preoperative CDVA was the only predictor for the postoperative vision, so appropriate patient selection, accurate preoperative measurements, and good intraoperative technique can result in excellent outcomes with minimal risk of complications.

Recent studies have shown promising results for correction of residual hyperopic or cylindrical refractive errors following cataract surgery using LAL technology (Calhoun Vision Inc.). The refractive power of the IOL can be adjusted and “locked in” postoperatively by the application of near-ultraviolet light [6, 7]. Also, because most cataract surgeons may not have equal access to a refractive laser, piggybacking may provide an excellent, yet cheaper, alternative to laser adjustment of IOL power in the eye [13].

Our study has some limitations including small patient cohort, its retrospective nature and the correct axis alignment, decentration, and tilt, which are major concerns, were only performed subjectively, and were not assessed with digital retroillumination imaging or ultrasound biomicroscopy. However, all 15 patients in this study were satisfied with the outcomes of the IOL exchange procedure, with the surgery improving or resolving their visual symptoms.

In spite of these limitations, our data suggest that piggybacking was an effective and predictable method for enhancing the refractive outcome and reducing spectacle dependence for distance in pseudophakic eyes.

## Figures and Tables

**Table 1:** The collected data for each case.

Case number	Age	Sex	Eye	Error SE	UDVA	CDVA	Duration of surgery	PMMA IOL power	Postop. SE	Postop. vision	Follow-up
1	63	f	Od	−2	6/24	6/9	5 m	−2	−0.25	6/9	26 m
2	56	f	Od	−3	6/36	6/9	7 m	−3	0.00	6/9	28 m
3	42	m	Od	−1.5	6/12	6/6	14 m	−1.5	0.00	6/6	24 m
4	68	m	Os	+3	6/60	6/12	4 m	+4.5	0.25	6/12	30 m
5	58	f	Od	−2.5	6/36	6/9	6 m	−2.5	0.00	6/9	28 m
6	56	m	Os	+2.5	6/60	6/9	18 m	+4	−0.5	6/9	29 m
7	49	f	Od	−4	6/36	6/9	1 m	−4	−0.25	6/9	36 m
8	70	f	Od	−7	5/60	6/18	12 m	−7	−0.1	6/18	33 m
9	54	m	Od	+3.5	5/60	6/9	6 m	+5.5	−0.5	6/9	27 m
10	64	f	Os	+2.5	6/60	6/12	5 m	+4	−0.25	6/12	34 m
11	66	f	Od	+4.5	4/60	6/12	12 m	+7	−0.5	6/12	26 m
12	59	f	Os	+3	6/60	6/9	16 m	+4.5	0.00	6/9	32 m
13	60	m	Os	+2	6/36	6/9	22 m	+3	0.00	6/9	29 m
14	50	m	Od	−3	6/36	6/6	30 m	−3	−0.25	6/6	24 m
15	55	m	os	+3	6/60	6/6	7 m	+4.5	−0.5	6/9	27 m

SE: spherical equivalent, UDVA: uncorrected distant visual acuity, CDVA: corrected distant visual acuity, and Postop.: postoperative.

**Table 2:** Patients demographic data.

	Range	Mean
Age	42–70	58 ± 7.60
Error	4.50 to −7.00	−0.23 ± 3.82
UDVA	0.30–1.18	0.88 ± 0.22
CDVA	0.00–0.48	0.19 ± 0.13
Duration	1.00–30.00	11 ± 7.88
IOL power	−7.00–7.00	0.93 ± 4.34
Refpost	−0.50 to 0.25	−0.19 ± 0.24
POSTVA	0.00–0.48	0.20 ± 0.12
Follow-up	24–36	28.88 ± 3.56

UDVA: uncorrected distant visual acuity, CDVA: corrected distant visual acuity, and Postop.: postoperative.

**Table 3:** Correlations.

		*r*	*P*
UCVA	Age	0.630	0.12
	CDVA	0.564	0.028
	IOL power	0.643	0.01
	Postop. VA	0.675	0.006

Error	IOL power	0.997	0.00

CDVA	Age	0.835	0.00
	UCVA	0.564	0.028
	Postop. VA	0.935	0.00
	Follow-up	0.582	0.023

Postop. VA	Age	0.869	0.00
	UDVA	0.675	0.006
	CDVA	0.935	0.000
	Follow-up	0.578	0.024

UDVA: uncorrected distant visual acuity, CDVA: corrected distant visual acuity, and Postop.: postoperative.
